# Acute Effects of Wearing Different Surgical Face Masks during High-Intensity, Short-Rest Resistance Exercise on Cardiorespiratory and Pulmonary Function and Perceptual Responses in Weightlifters

**DOI:** 10.3390/biology11070992

**Published:** 2022-06-29

**Authors:** Shin-Yuan Wang, Chih-Hui Chiu, Chin-Hsien Hsu, Chun-Chung Chou, Shuo-Min Hsu, Lu-Bi Shapu, Tai-Chen Chao, Che-Hsiu Chen

**Affiliations:** 1 Department of Exercise Health Science, National Taiwan University of Sport, Taichung 404401, Taiwan; wowbat0623@ntus.edu.tw (S.-Y.W.); chiuch@ntus.edu.tw (C.-H.C.); 2 Department of Leisure Industry Management, National Chi-Yi University of Technology, Taichung 411030, Taiwan; 3 Physical Education Office, National Taipei University of Technology, Taipei 10608, Taiwan; longer0206@gmail.com; 4 Cheng Ching Hospital, Taichung 407211, Taiwan; u103022415@cmu.edu.tw; 5 School of Physical & Health, Nanning Normal University, Nanning 530011, China; bikeprince@hotmail.com; 6 Sport Science Research Center, National Taiwan University of Sport, Taichung 404401, Taiwan; ted830820@ntus.edu.tw; 7 Department of Sport Performance, National Taiwan University of Sport, Taichung 404401, Taiwan

**Keywords:** postexercise hypotension, resistance training, face mask, ventilation

## Abstract

**Simple Summary:**

Wearing a face mask can block and reduce the exposure of the oronasopharyngeal region to viruses. It is unclear whether wearing a surgical mask (SM) or a three-dimensional (3D) SM (3DSM) during high-intensity, short-rest resistance exercise could influence the cardiac capacity, pulmonary function, and comfort in weightlifters. Wearing both SM and 3DSM during whole-body, high-intensity, short-rest resistance exercise exerted no detrimental effect on blood pressure (BP) or pulmonary function and promoted postexercise hypotension (PEH). Furthermore, wearing a typical SM during exercise produced higher breathing resistance and tightness than did wearing a 3DSM or no mask.

**Abstract:**

This study investigated the effect of wearing a typical surgical mask (SM) or a three-dimensional (3D) SM (3DSM) during whole-body, high-intensity, short-rest resistance exercise on cardiorespiratory, respiratory, and perceptual comfort responses in weightlifters. Twenty elite weightlifters (6 women and 14 men; age = 24.1 ± 4.9 years; height: 167.45 ± 7.60 cm; body mass = 76.48 ± 19.86 kg) who participated in this study performed 3 resistance exercise sessions in a randomized order: (1) without a mask (NM), (2) while wearing a typical SM, and (3) while wearing a 3DSM. Resistance exercise consisted of a descending pyramid scheme starting at 10 repetitions, with a decrease of one repetition per set for the back squat, bench press, and deadlift, as fast as possible at 75% of the one-repetition maximum. Cardiorespiratory and pulmonary function and comfort were measured. Across all conditions, effective postexercise hypotension (PEH) was noted in terms of decreased systolic blood pressure (−4.64%), diastolic BP (−5.36%), mean arterial pressure (−5.02%), and ankle–brachial index (−6.84%). However, the heart rate (40.34%) and rate of pressure product (33.60%) increased, and no effects on pulmonary function were observed in the three conditions. The participants reported higher breathing resistance and tightness when wearing a typical SM than when wearing a 3DSM or no mask. Therefore, both wearing and not wearing a face mask during whole-body, high-intensity, short-rest resistance exercise promoted PEH and exerted no detrimental effect on pulmonary function. Coaches, trainers, and athletes should consider wearing a 3DSM during resistance exercise.

## 1. Introduction

COVID-19 has caused numerous deaths and confirmed cases worldwide [1]. COVID-19 is transmitted by droplets and causes upper respiratory tract disease and acute respiratory distress syndrome. However, a study investigating the relationship between exercise habits and disease severity in 48,440 patients with COVID-19 infection reported that the patients who were physically inactive (<10 min of physical activity per week) had higher rates of hospitalization (2.26 times), admission to the intensive care unit (1.73 times), and death (2.49 times) than did those who exercised at least 150 minutes per week. Therefore, individuals should be physically active [2]. However, the Center for Disease Control and American College of Sports Medicine recommend engaging in moderate- to high-intensity exercise to maintain cardiorespiratory and muscular fitness [1] and enhance immune function [3].

Wearing a face mask can block and reduce the exposure of the oronasopharyngeal region to viruses. However, in June 2020, the World Health Organization released a statement indicating that people should not wear a mask during exercise because it may reduce their ability to breathe comfortably [4] and pulmonary function [5]. However, the effects of wearing a face mask during different types of exercise on physiological and athletic performance remain unclear.

A study reported that wearing a surgical mask (SM) or an N95 face mask during aerobic exercise reduced ventilation, exercise time capacity (only for N95 masks), and comfort during a stepwise incremental cycle ergometer test [5]. Another study demonstrated that wearing an SM or N95 face mask during aerobic exercise reduced the maximum performance, minute ventilation, and oxygen consumption [6]. In addition, wearing a cloth mask during a maximal cardiopulmonary exercise test on a treadmill significantly reduced exercise time, maximal oxygen consumption, and comfort [7]. Similarly, wearing a SM during a walking test on a treadmill (40 minutes, with a starting speed of 4.5 km/h and an ending speed being of 6 km/hour) reduced oxygen delivery, ventilation, and heart rate (HR), and significantly increased blood pressure (BP) during exercise [8]. However, controversial findings have also been reported [9,10]. For example, compared with not wearing any mask, wearing an SM or an N95 face mask during a stepwise incremental cycle ergometer test did not significantly reduce exercise time and physiological parameters (including the HR, respiratory rate, BP, and oxygen saturation [SpO_2_]) [9]. Similarly, wearing an SM or cloth mask during a cycle ergometer test had no effect on blood or muscle oxygenation and exercise performance [10].

In terms of resistance exercise, wearing an N95 face mask reduced the mean and maximum propulsive velocity during high-intensity (70% one-repetition maximum [RM]) bench press until failure, and SpO_2_ and increased the perceived exertion rating during moderate-intensity exercise (50% one-RM) in recreational weight lifters [11]. However, the study suggested that during resistance training exercise (4 sets of 10 repetitions of a half-squat at 60% of the one-RM), the strength performance and physiological responses (HR, HR variability, and blood lactate concentration) observed when wearing an SM or an FFP2 face mask were similar to those observed without a mask in patients with sarcopenia [12].

High-intensity cross-training programs (such as CrossFit) have become particularly popular because they combine changing exercise intensities and various workouts with high intensity and short- or no-rest periods to perform cyclic total body resistance training movements that can enhance aerobic fitness and reduce body fat, [13], develop muscle power and endurance [14], and increase metabolic capacity [15].

During resistance exercise, several acute changes can occur in the cardiovascular system, including increases in the HR, rate pressure product (RPP), and BP. These acute cardiovascular responses are affected by exercise intensity, repetition, exercise type, and muscle contraction type [16,17]. For example, although high-intensity resistance training (3 sets of 10 repetitions at 75% one-RM for the squat, bench press, and deadlift) exerted no effect on brachial or aortic BP, it immediately increased aortic arterial stiffness [18,19]. Similarly, Shaw et al. (2015) reported significant increases in HR, RPP, and blood lactate levels but a decrease in forced expiratory 1 second (FEV1) after 10 minutes of CrossFit exercise (consisting of three burpees, four push-ups, and five squats). However, CrossFit had no effect on BP parameters (systolic BP [SBP], diastolic BP [DBP], and mean arterial pressure [MAP]) or pulmonary function parameters (forced vital capacity [FVC] and FEV1/FVC [%]) [20]. By contrast, Dantas et al. (2018) observed significantly decreased SBP, DBP, and MAP after CrossFit exercise (consisting of 20 kettlebell reps, 15 wallballs, and 50 doubleunders for 1 minute, and each set of exercises was repeated in seven rounds) [17] this is known as “postexercise hypotension” (PEH). Thus, the effects of immediate cardiovascular and pulmonary changes occurring after resistance training remain unknown.

To the best of our knowledge, the effect of wearing an SM or a three-dimensional (3D) SM (3DSM) during high-intensity, short-rest resistance exercise on the cardiac capacity, pulmonary function, and comfort has not yet been analyzed. Therefore, this study investigated the effect of wearing a typical SM or 3DSM during whole-body, high-intensity, short-rest resistance exercise on cardiorespiratory, respiratory, and perceptual comfort responses in weightlifters. We hypothesized that wearing a face mask during exercise, regardless of the mask type, would considerably affect the HR, brachial–ankle pulse wave velocity (baPWV), ankle–brachial index (ABI), SBP, DBP, RPP, MAP, SpO_2_, pulmonary function, and comfort in weightlifters.

## 2. Materials and Methods

### 2.1. Experimental Design

**Figure 1:**
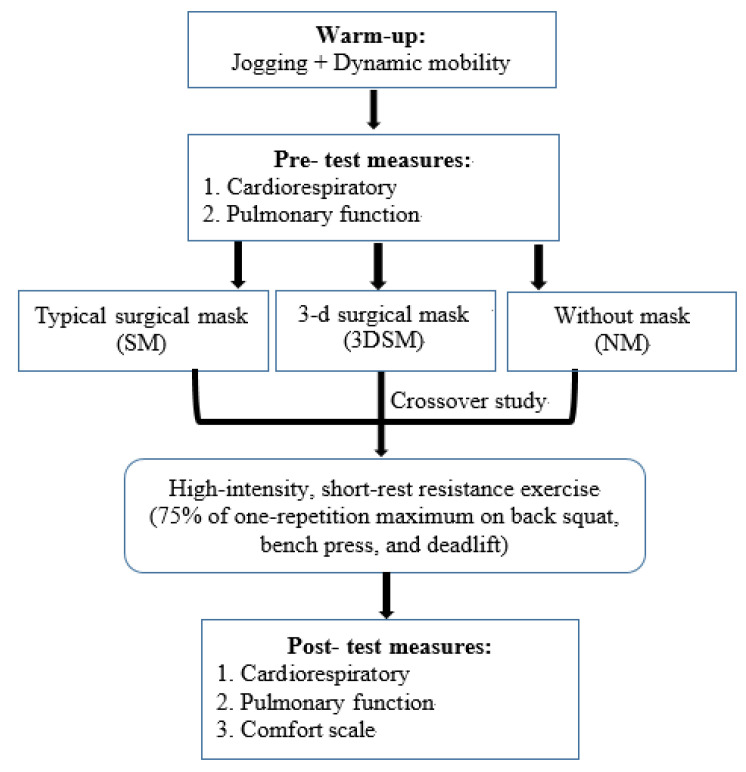
Schematic representation of the study design and procedure.

Participants visited the laboratory 4 times. One week before the first experimental testing visit, all participants were familiarized with the procedures and underwent one-RM testing. Then, 3 whole-body, high-intensity, short-rest resistance exercise tests were performed every 7 days in a randomized order: without a mask (NM), with a typical SM, and with a 3DSM. During each experimental visit, participants completed one warmup protocol (jogging for 5 min on a treadmill at 6.4 km·h^−1^ on a 1% incline and dynamic mobility), and the subsequent measurement tests were performed. Figure 1 shows the experimental design of the study.

### 2.2. Participants

A total of 20 elite weightlifter athletes (women: *n* = 6; men: *n* = 14; age = 24.1 ± 4.9 years; height: 167.45 ± 7.60 cm; body mass = 76.48 ± 19.86 kg) volunteered to participate in this study. On average, they trained for 2 to 3 hours (including resting periods) a day 5 times a week. All participants were categorized in National Division I. Those who experienced lower-extremity injury in the year prior, lower back pain, and cardiovascular diseases were excluded from this study. Each participant provided informed consent before testing. All participants refrained from participating in vigorous physical activities and training at least 3 days before any experimental visit. On all experimental visit days, all participants refrained from consuming alcohol and caffeine for at least 12 h. All experimental procedures were performed in accordance with the Declaration of Helsinki and were approved by the Institutional Review Board of Jen-Ai Hospital (approval number: IRB-109-94).

### 2.3. Face Masks

We used a three-ply general surgical face mask (low fluid resistance: 2.06 mmH_2_O/cm^2^; ACTife HI-TECH CO., LTD., New Taipei City, Taiwan), and a novel 3-dimensional surgical face mask (low fluid resistance: 2.06 mmH_2_O/cm^2^; ACTife HI-TECH CO., LTD, New Taipei City, Taiwan). To ensure appropriate breathing, this 3D face mask tents high and wide off the face. The spirometry mask was placed over the NM, SM, and 3DSM and fixed in place with head straps to prevent air leaks.

### 2.4. Measurement of Dependent Variables

All dependent variables were measured after general warmup (pre-test) and immediately after resistance exercise (post-test) in the following order: HR, baPWV, ABI, SBP, DBP, RPP, MAP, SpO_2_, pulmonary function, and comfort.

### 2.5. BP, baPWV, HR, and SpO_2_

An automatic waveform analyzer (VP-1000, Omron Healthcare Co., Ltd., Kyoto, Japan) was used to measure SBP, DBP, baPWV, and ABI simultaneously during the pre-test and post-test [21]. The participants were instructed to quietly rest in the supine position for at least 10 minutes. Occlusion and cuffs were wrapped around the bilateral brachial and tibial arteries in accordance with the method reported in another study [21]. The HR was collected using a heart rate monitor (Polar V800, Electro Oi, Kempele, Finland). Continuous peripheral blood SpO_2_ measurements were performed using a wearable finger pulse oximeter (SA-310, Rossmax, Jubei, Hsinchu, Taiwan). MAP was calculated using the formula (SBP + [2DBP])/3 [22]. RPP is considered a valid predictor of cardiovascular risk. RPP was calculated using the index of myocardial oxygen consumption as follows: RPP = HR × SBP [23,24].

### 2.6. Pulmonary Function Parameters

Pulmonary function was examined using the Master Screen Pneumo (San Diego, CA, USA) to measure FVC, FEV_1_, tidal volume (TV), FEV/FVC (%), and maximum voluntary ventilation (MVV) in accordance with the American Thoracic Society and European Respiratory Society guidelines [25].

### 2.7. Comfort/Discomfort Scale

The comfort and discomfort scale consisted of 10 domains: breathing resistance, tightness, feeling unfit, humidity, heat, odor, fatigue, itchiness, saltiness, and overall discomfort. Immediately after the mask-wearing conditions, the participants were asked to indicate their comfort and discomfort by using a visual analog scale ranging from 0 (not at all) to 10 (strong discomfort) [26].

### 2.8. Whole-Body High-Intensity Short-Rest Resistance Exercise

The exercise protocol consisted of 3 barbell exercises, back squats, bench presses, and deadlifts. The first set of the exercise protocol consisted of 10 repetitions of each of the 3 barbell exercises. Each progressive set decreased by one repetition and finished with the final set of one repetition (descending pyramid fashion). The weight was set at 75% one-RM for each exercise. The participants were asked to complete the sets and repetitions as fast as possible, with minimal rest in between sets and exercises [15].

### 2.9. Statistical Analysis

Data are presented as the mean ± standard deviation. The variables that passed the Shapiro–Wilk test for normality were analyzed using SPSS (version 25; IBM, Armonk, NY, USA). A separate 2-way (time: pre-test vs. post-test) × 3 (conditions: NM vs. SM vs. 3DSM) repeated-measures analysis of variance (ANOVA) was performed to analyze data. When appropriate, follow-up tests, namely a one-way repeated-measures ANOVA with Bonferroni-adjusted pairwise comparisons and the paired sample *t* test, were performed. Statistical significance was *p* < 0.05. The effect size (ES) was calculated using partial eta squared (η^2^). The ES was considered small if η^2^ < 0.06 and large if η^2^ > 0.14.

## 3. Results

The ANOVA tests showed no statistical significances (*p* > 0.05) for time × intervention interaction on all dependent variables. In addition, The ANOVA tests showed statistical significances (*p* < 0.05) for intervention conditions on breathing resistance and tightness scale.

### 3.1. BP, baPWV, HR, and SpO_2_

**Figure 2:**
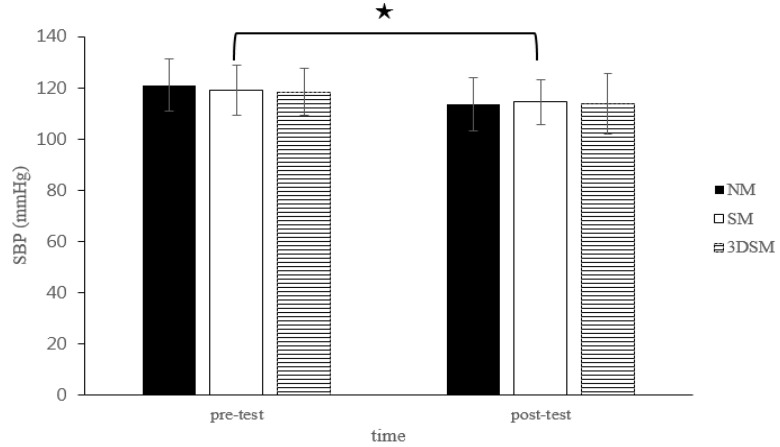
Changes in SBP after without mask, wearing an SM or 3DSM during high-intensity, short-rest resistance exercise. ^★^ Significant difference between different time points for combined mean values (*p* < 0.05).

At the pretest, no significant differences in the dependent variables were noted among the three conditions. Table 1 lists results for the HR, RPP, SBP, DBP, MAP, SpO_2_, baPWV, and ABI. For SBP, the analysis did not indicate a two-way time × intervention interaction (F = 1.89; *p* = 0.17; η^2^ = 0.09) or the main effect of condition (F = 0.80; *p* = 0.46; η^2^ = 0.04). However, a significant main effect of time was noted (F = 30.71; *p* < 0.001; η^2^ = 0.62). Across the conditions, SBP significantly decreased from the pretest to posttest (Figure 2).

**Figure 3:**
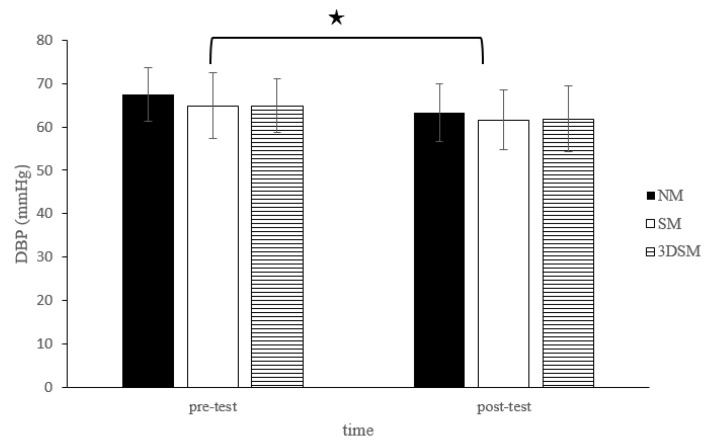
Changes in DBP after without mask, wearing an SM or 3DSM during high-intensity, short-rest resistance exercise. ^★^ Significant difference between different time points for combined mean values (*p* < 0.05).

For DBP, the analysis did not reveal a two-way time × intervention interaction (F = 0.54; *p* = 0.59; η^2^ = 0.03); however, a significant main effect of time (F = 18.07; *p* < 0.001; η^2^ = 0.49) and the main effect of condition (F = 4.63; *p* = 0.02; η^2^ = 0.20) were noted. Across the conditions, DBP significantly decreased from the pretest to posttest. Across time, the SM and 3DSM conditions resulted in significantly lower DBP than did the NM condition (Figure 3).

**Figure 4:**
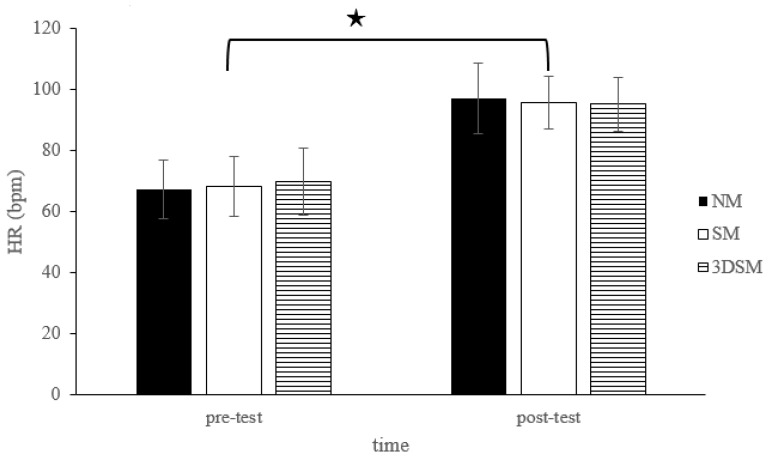
Changes in HR after without mask, wearing an SM or 3DSM during high-intensity, short-rest resistance exercise. ^★^ Significant difference between different time points for combined mean values (*p* < 0.05).

For the HR, the analysis did not indicate a two-way time × intervention interaction (F = 1.97; *p* = 0.15; η^2^ = 0.09) or the main effect of condition (F = 0.03; *p* = 0.97; η^2^ = 0.002). However, a significant main effect of time was noted (F = 233.68; *p* < 0.001; η^2^ = 0.93). Across the conditions, the HR significantly increased from the pretest to the posttest (Figure 4).

**Figure 5:**
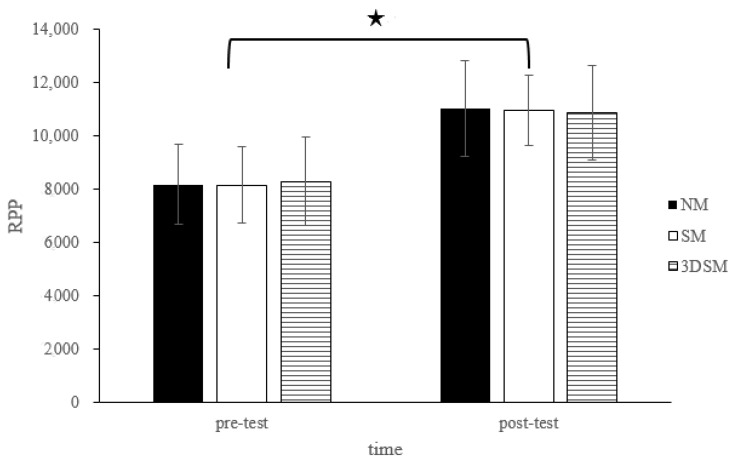
Changes in RPP after without mask, wearing an SM or 3DSM during high-intensity, short-rest resistance exercise. ^★^ Significant difference between different time points for combined mean values (*p* < 0.05).

For the RPP, the analysis did not reveal a two-way time × intervention interaction (F = 0.48; *p* = 0.62; η^2^ = 0.03) or the main effect of condition (F = 0.02; *p* = 0.98; η^2^ = 0.001). However, a significant main effect of time was noted (F = 110.80; *p* < 0.001; η^2^ = 0.85). Across the conditions, the RPP significantly increased from the pretest to posttest (Figure 5).

**Figure 6:**
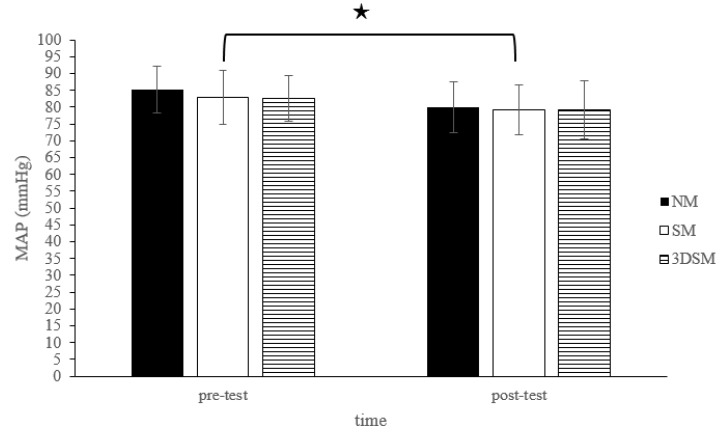
Changes in MAP after without mask, wearing an SM or 3DSM during high-intensity, short-rest resistance exercise. ^★^ Significant difference between different time points for combined mean values (*p* < 0.05).

For the MAP, the analysis did not reveal a two-way time × intervention interaction (F = 1.29; *p* = 0.29; η^2^ = 0.06) or the main effect of condition (F = 3.06; *p* = 0.06; η^2^ = 0.14). However, a significant main effect of time was noted (F = 27.46; *p* < 0.001; η^2^ = 0.59). Across the conditions, the MAP significantly decreased from the pretest to posttest (Figure 6).

**Figure 7:**
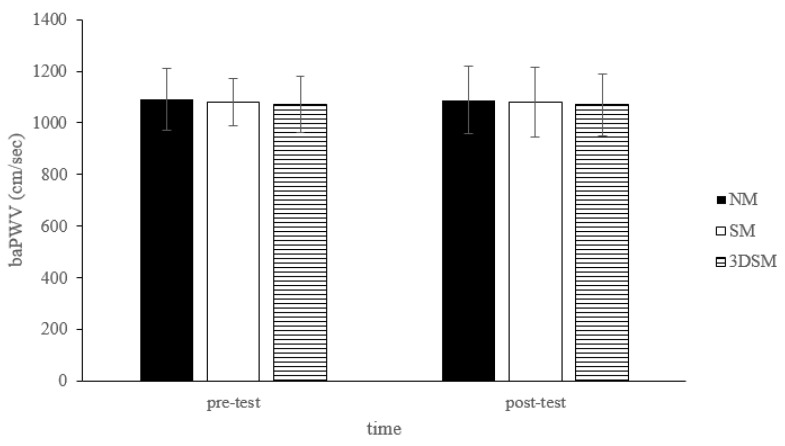
Changes in baPWV after without mask, wearing an SM or 3DSM during high-intensity, short-rest resistance exercise.

For baPWV, the analysis did not reveal a main effect of condition (F = 1.18; *p* = 0.32; η^2^ = 0.06), time (F = 0.01; *p* = 0.91; η^2^ = 0.001), or a condition × time interaction (F = 0.04; *p* = 0.97; η^2^ = 0.002) (Figure 7).

**Figure 8:**
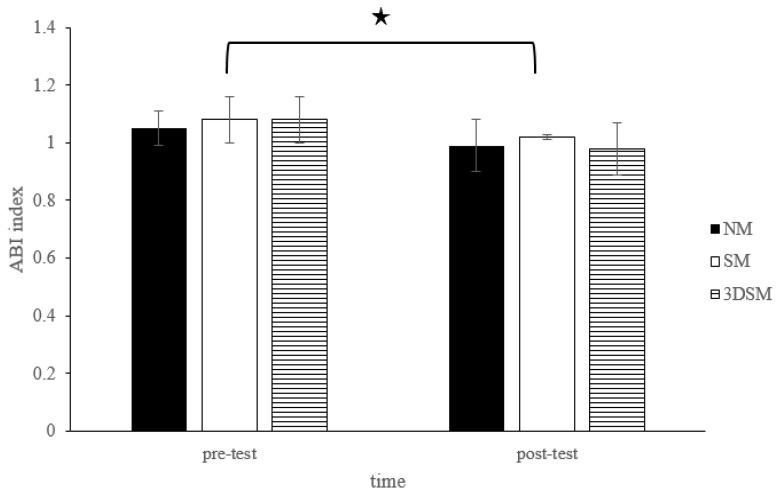
Changes in ABI after without mask, wearing an SM or 3DSM during high-intensity, short-rest resistance exercise. ^★^ Significant difference between different time points for combined mean values (*p* < 0.05).

For the ABI, the analysis did not exhibit a two-way time × intervention interaction (F = 1.15; *p* = 0.33; η^2^ = 0.06) or a main effect of condition (F = 0.80; *p* = 0.46; η^2^ = 0.04). However, a significant main effect of time (F = 27.71; *p* < 0.001; η^2^ = 0.59) was noted. Across the conditions, the ABI significantly decreased from the pretest to posttest (Figure 8).

For SpO_2_, the analysis did not indicate a main effect of condition (F = 0.43; *p* = 0.66; η^2^ = 0.02), time (F = 3.43; *p* = 0.08; η^2^ = 0.15), or a condition × time interaction (F = 1.15; *p* = 0.33; η^2^ = 0.06).

### 3.2. Pulmonary Function Parameters

Table 2 lists the results for FVC, FEV_1_, TV, FEV/FVC (%), and MVV. For the FVC, the analysis indicated no main effect of condition (F = 1.12; *p* = 0.34; η^2^ = 0.06), time (F = 0.01; *p* = 0.93; η^2^ = 0.00), or a condition × time interaction (F =2.82; *p* = 0.07; η^2^ = 0.13). For the FEV_1_, the analysis revealed no main effect of condition (F = 0.89; *p* = 0.42; η^2^ = 0.05), time (F = 1.26; *p* = 0.28; η^2^ = 0.06), or a condition × time interaction (F = 2.75; *p* = 0.08; η^2^ = 0.13). For the TV, the analysis revealed no main effect of the condition (F = 3.17; *p* = 0.05; η^2^ = 0.14), time (F = 3.92; *p* = 0.06; η^2^ = 0.17), or a condition × time interaction (F = 0.05; *p* = 0.95; η^2^ = 0.003). For the FEV/FVC (%), the analysis revealed no main effect of the condition (F = 0.15; *p* = 0.86; η^2^ = 0.008), time (F = 6.87; *p* = 0.02; η^2^ = 0.27), or a condition × time interaction (F = 1.97; *p* = 0.15; η^2^ = 0.09). For the MVV, the analysis revealed no main effect of the condition (F = 2.25; *p* = 0.12; η^2^ = 0.11), time (F = 1.15; *p* = 0.30; η^2^ = 0.06), or a condition × time interaction (F = 0.51; *p* = 0.60; η^2^ = 0.03).

### 3.3. Perceived Exertion and Comfort/Discomfort Scale

Table 3 lists the results for posttrial perceived exertion and the comfort/discomfort scales. Breathing resistance (F = 5.75; *p* = 0.005) and tightness (F = 8.88; *p* < 0.001) significantly differed among the three conditions and were significantly higher scale in the SM condition than in the NM and 3DSM conditions. In addition, no significant difference was noted in humidity (F = 0.42; *p* = 0.66), heat (F = 0.01; *p* = 0.99), itchiness (F = 0.38; *p* = 0.69), saltiness (F = 0.17; *p* = 0. 84), feeling unfit (F = 1.56; *p* = 0.22), odor (F = 1.63; *p* = 0.20), fatigue (F = 0.58; *p* = 0.56), and overall discomfort (F = 0.62; *p* = 0.54) for the three conditions.

## 4. Discussion

To the best of our knowledge, this study is among the few to investigate the effects of wearing a typical SM or 3DSM during whole-body, high-intensity, short-rest resistance exercise on the cardiorespiratory, pulmonary function, and perceptual responses of the weightlifters. The main finding is that wearing a typical SM or 3DSM during whole-body, high-intensity, short-rest resistance exercise did not exert a detrimental effect on BP and even reduced BP, MAP, and ABI after whole-body, high-intensity, short-rest resistance exercise. However, the HR and RPP increased in all the conditions. The pulmonary function parameters were unaffected in all three conditions. Wearing a typical SM was considerably uncomfortable and exerted a marked effect on subjective breathing resistance and tightness. In addition, compared with a 3DSM, a typical SM led to severe subjective discomfort in breathing resistance and tightness.

### 4.1. Cardiac Function

The effects of wearing different types of surgical face masks during whole-body, high-intensity, short-rest resistance exercise on cardiac function (HR, RPP, SBP, DBP, MAP, baPWV, and ABI) remain unclear, and the results of other studies are inconsistent. In our study, the three conditions did not affect cardiac function differently. Across the conditions, SBP (−4.64%), DBP (−5.36%), MAP (−5.02%), and ABI (−6.84%) significantly decreased from the pretest to posttest.

However, different from our results, one study revealed that SBP significantly increased in weightlifters who wore an FFP2 or N95 mask during both 50% and 70% one-RM intensity upper-body resistance exercise (four sets in the bench press exercise performed until movement failure).

However, DBP significantly increased only in the 70% one-RM intensity condition. In addition, lower SpO_2_ and higher RPE were noted in participants who wore a mask than in those who did not wear a mask in the 50% one-RM intensity condition, and no significant difference in the HR was observed between the intensity conditions [11]. Similarly, a study observed higher SBP (rest = 128 mmHg to exhaustion = 227 mmHg) after bicycle ergometer incremental exercise tests in athletes who wore an SM or FFP2 mask or did not wear a face mask and noted no difference in the HR between the conditions [6]. In addition, a study noted significantly higher SBP (rest =106 mmHg to the final exercise stage = 227 mmHg) and DBP (rest = 61 mmHg to the final exercise stage = 74 mmHg) after a maximal cardiopulmonary exercise test on a treadmill in participants who wore or did not wear a cloth face mask [7].

Wearing an SM during exercise did not lead to the deterioration of cardiopulmonary function [6,7,12,27]. A study demonstrated that wearing an SM or FFP2 mask during lower-body resistance training (4 sets of 10 repetitions of a half-squat at 60% one-RM) did not result in significant differences in the HR, HR variability, blood lactate levels, and RPE among the three conditions (without a mask, with an SM, and with an FFP2 mask) [12]. In addition, compared with not wearing a face mask, wearing an SM significantly increased the HR and airway resistance during a constant-load cycle ergometer test, and BP and RPE did not differ between those wearing and those not wearing a mask [27].

However, the blood pressure and arterial stiffness index responses observed after high-intensity, short-rest resistance exercise in our study are inconsistent with those reported in other studies. For example, studies have indicated that high-intensity resistance training (three sets of 10 repetitions at 75% one-RM for a squat, bench press, and deadlift, with 2 minutes of rest between sets and exercises; the total volume was 90 repetitions × 75% one-RM) significantly increased the HR and exerted no effect on brachial or aortic BP; however, it immediately increased aortic arterial stiffness [18,19].

Similarly to other studies, this study demonstrated the occurrence of PEH after resistance exercise [28,29,30,31]. However, hypotension after resistance exercise is associated with larger muscle groups (e.g., the lower extremity), training volume, [28,29], and training intensity [30,31]. The training volume (sets × repetitions × load × exercises) was more crucial than the intensity itself to induce PEH [32].

Simão et al. (2005) reported that resistance training (three sets of 6-RM of 5 and 6 exercises) significantly reduced SBP from pre-exercise to 50 minutes after resistance training. DBP significantly decreased at 10 minutes after the three sets of 12 repetitions of 6 exercises [29]. In addition, the study revealed that both whole-body high-intensity resistance exercise protocols (three sets of one session at 40% and 80% one-RM for the leg press, leg extension, leg curl, chest press, elbow flexion, elbow extension, upper back row, and abdominal flexion) were similarly effective in inducing PEH [32]. However, studies have indicated that SBP, DBP, and MBP significantly decreased after both 50% and 80% one RM exercises consisting of a set of 10 repetitions of 10 exercises [30,31].

Furthermore, PEH was higher at 80% one-RM than at 50% one-RM, and the intensity of 80% one-RM considerably increased forearm blood flow, HR, vasodilator responses, [30,31], and cardiac sympathovagal balance [31], Increased blood flow [31], HR [30], and nitric oxide release from the vascular endothelium [33] after resistance exercise might be responsible for PEH or a decrease in aortic arterial stiffness. One study reported that the nitric oxide level increased after resistance training (at 70% one-RM) and power training (at 50% one-RM) involving 3 sets of 8–10 repetitions of 8 exercises and that SBP decreased after power training. [33]. Resistance training performed for large muscle groups or consisting of numerous sets may increase blood flow and cause the release of more vasoactive substances [28]. Polito and Farinatti (2009) compared PEH following the biceps curl and leg extension with that following different numbers of sets (6 or 10 sets of 10 repetitions at 12 RM) and reported that PEH (decreased SBP and MAP) was observed only for the leg group when 10 sets were performed [28]. In addition, both cardio–ankle vascular index and baPWV were lower for lower-body resistance training than for upper-body resistance training (4 sets of 10 repetitions at 70% one RM) [34].

Noteworthy, our results suggest that, whole-body, high-intensity, short-rest resistance exercise performed with or without an SM did not adversely affect cardiopulmonary function and promoted PEH in weightlifters.

### 4.2. Pulmonary Function

Regarding pulmonary function, our results reveal that wearing an SM during resistance training did not exert negative effects on SpO_2_, FVC, FEV1, TV, FEV/FVC (%), and MVV. This finding is inconsistent with those of other studies. For a nonmaximal exercise test, a study observed no negative effect of wearing a cloth mask or an SM on CO_2_ and SpO_2_ during sitting quietly or walking briskly [35]. A study using an incremental exertion exercise test demonstrated that wearing an SM or a cloth mask exerted no negative effect on arterial oxygen saturation and tissue oxygenation index during a cycle ergometry test performed until exhaustion [10]. By contrast, Fikenzer et al. (2020) reported that wearing an SM or an FFP2/N95 mask during incremental exertion exercise significantly reduced FVC, FEV1, and peak expiratory flow [5]. In addition, wearing an SM or FFP2 mask during bicycle ergometer incremental exertion exercise affected peak minute ventilation and oxygen consumption in well trained athletes [6]. The inconsistency in the findings could be due to the cardioventilatory response being greater in aerobic exercise than in resistance training [12,36]. However, Albesa-Albiol et al. (2019) reported that the oxygen uptake (VO_2_) was significantly higher in cycle ergometery endurance exercise than half-squat resistance exercise [36]. In addition, previous studies have reported that inspiratory muscle fatigue occurs after a variety of aerobic exercises [37,38,39,40]. Therefore, we speculate that wearing a face mask during aerobic exercise would have a greater negative effect on pulmonary function than resistance exercise.

### 4.3. Comfort/Discomfort Scale

Our results reveal that breathing resistance and tightness were higher in the SM condition than in the NM and 3DSM conditions. No significant differences in humidity, heat, itchiness, saltiness, feeling unfit, odor, fatigue, and overall discomfort were observed among the three conditions. A study reported that wearing an SM or FFP2 mask did not affect the rating of perceived exertion during lower-extremity resistance exercise (4 sets of 10 repetitions of half-squats at 60% one-RM) [12].

By contrast, Rosa et al. (2021) revealed that wearing an FFP2 or N95 face mask significantly increased the rating of perceived exertion during moderate-resistance exercise (four sets of bench press until movement failure at 50% one-RM) [11]. Another study reported that wearing an SM increased the rating of perceived exertion only when the intensity reached a high level (75% maximal oxygen uptake) and caused more discomfort in terms of heat, humidity, and breathing resistance [41]. In addition, the use of an SM or FFP2/N95 mask led to increased discomfort in terms of breathing resistance, tightness, feeling unfit, heat, fatigue, itchiness, and overall discomfort [5]. Egger et al. (2021) indicated that most participants (69%) reported acute dyspnea from the suction of the wet and deformed SM and that few participants (13%) reported acute dyspnea when wearing a FFP2 mask, most likely because of the 3D design of the FFP2 [6], This finding is consistent with our results indicating higher breathing resistance and tightness with an SM than with an NM or 3DSM.

This study has some limitations that should be considered when the results are interpreted. The study population consisted of well-trained weightlifter athletes; thus, the results cannot be generalized to untrained persons, older people, or those with hypertension.

## 5. Conclusions

Wearing both SMs and 3DSMs during whole-body, high-intensity, short-rest resistance exercise exerted no detrimental effect on BP and pulmonary function and even reduced BP, MAP, and ABI. However, wearing a typical SM during high-intensity, short-rest resistance exercise produced higher breathing resistance and tightness than did wearing a 3DSM or no mask. During the COVID-19 pandemic, a mask should be worn to perform resistance exercise training. Because a 3DSM is more comfortable than a typical SM, 3DSM masks are preferable.

## Figures and Tables

**Table 1:** Mean ± standard deviation of HR, RPP, SBP, DBP, MAP, SpO_2_, baPWV, and ABI following each condition.

	NM		SM		3DSM		*p*
	Pre Test	Post Test	Pre Test	Post Test	Pre Test	Post Test	
SBP (mmHg)	121.05 ± 10.19	113.55 ± 10.33	119.00 ± 9.99	114.40 ± 8.91	118.35 ± 9.23	113.80 ± 11.73	0.17
DBP (mmHg)	67.45 ± 6.21	63.20 ± 6.60	64.85 ± 7.56	61.60 ± 6.83	64.90 ± 6.27	61.80 ± 7.61	0.59
HR (bpm)	67.30 ± 9.50	97.05 ± 11.70	68.35 ± 9.84	95.85 ± 8.65	69.70 ± 11.06	95.20 ± 8.85	0.15
RPP	8172.00 ± 1509.74	11,030.60 ± 1779.71	8146.85 ± 1430.19	10,966.35 ± 1320.17	8281.90 ± 1665.14	10,865.95 ± 1763.83	0.62
MAP (mmHg)	85.32 ± 6.93	79.99 ± 7.49	82.90 ± 7.96	79.20 ± 7.30	82.72 ± 6.81	79.13 ± 8.69	0.29
SpO2 (%)	97.30 ± 0.93	96.80 ± 1.36	97.25 ± 0.85	97.15 ± 0.88	97.20 ± 1.00	96.95 ± 0.89	0.33
baPWV (cm/sec)	1091.25 ± 119.63	1087.20 ± 130.90	1078.85 ± 91.91	1080.30 ± 135.99	1071.55 ± 107.46	1070. 10 ± 121.00	0.97
ABI index	1.05 ± 0.06	0.99 ± 0.09	1.08 ± 0.08	1.02 ± 0.01	1.08 ± 0.08	0.98 ± 0.09	0.33

NM: without mask; SM: surgical mask; 3DSM: 3-D surgical mask. SBP: systolic blood pressure; DBP: diastolic blood pressure; HR: heart rate. RPP: rate pressure product; MAP: mean arterial pressure; SpO_2_: blood oxygen saturation; baPWV: brachial ankle pulse wave velocity; ABI index: ankle–brachial index.

**Table 2:** Mean ± standard deviation of FVC, FEV_1_, TV, FEV/FVC (%), and MVV following each condition.

	NM		SM		3DSM		*p*
	Pre Test	Post Test	Pre Test	Post Test	Pre Test	Post Test	
FVC (l)	3.87 ± 0.69	3.90 ± 0.74	3.99 ± 0.64	3.90 ± 0.58	3.86 ± 0.72	3.90 ± 0.65	0.07
FEV_1 (l)_	3.60 ± 0.61	3.69 ± 0.67	3.72 ± 0.59	3.69 ± 0.57	3.62 ± 0.66	3.67 ± 0.59	0.08
TV (l)	0.88 ± 0.35	0.97 ± 0.38	1.10 ± 0.43	1.19 ± 0.52	1.04 ± 0.37	1.11 ± 0.49	0.95
FEV/FVC (%)	92.56 ± 6.83	94.44 ± 5.96	93.26 ± 7.62	94.44 ± 7.14	93.57 ± 6.35	93.95 ± 6.44	0.15
MVV (L/min)	134.19 ± 30.66	135.37 ± 30.55	144.65 ± 33.46	145.01 ± 30.23	137.34 ± 38.75	141.56 ± 33.50	0.60

FVC: forced vital capacity; FEV_1_: forced expiratory volume in 1st second; TV: tidal volume; MVV: Maximum voluntary ventilation.

**Table 3:** Mean ± standard deviation of perceived exertion and comfort/discomfort scale following each condition.

	NM	SM	3DSM	*F*	*p*
Humidity	5.95 ± 2.65	5.48 ± 2.18	5.22 ± 2.77	0.42	0.67
Heat	5.18 ± 2.77	5.13 ± 2.38	5.05 ± 2.74	0.01	0.99
Breathing resistance	3.55 ± 1.90 *	5.25 ± 1.78	3.50 ± 1.88 *	4.37	0.005
Itchiness	1.27 ± 1.92	1.13 ± 1.41	1.60 ± 1.89	0.38	0.69
Tightness	2.52 ±1.42 *	4.13 ± 2.57	1.60 ± 1.53 *	8.88	<0.001
Saltiness	1.48 ± 1.99	1.20 ± 1.50	1.55 ± 2.33	0.17	0.84
Feeling unfit	4.15 ± 2.08	4.53 ± 1.87	3.32 ± 2.57	1.56	0.22
Odor	1.23 ± 1.52	2.08 ± 1.88	2.23 ± 2.19	1.63	0.20
Fatigue	4.50 ± 2.09	4.68 ± 2.35	3.88 ± 2.69	0.62	0.54
Overall discomfort	4.78 ± 1.63	4.98 ± 1.83	4.35 ± 2.12	0.58	0.56

* Significant differences between NM and SM and between 3DSM and SM.

## Data Availability

The data presented in this study are available on request from the corresponding author. The data are not publicly available due to this not being included in the Institutional Ethics Committee approval.

## References

[1] Chandrasekaran B., Fernandes S. (2020). “Exercise with facemask; Are we handling a devil’s sword?”—A physiological hypothesis. Med. Hypotheses.

[2] Sallis R., Young D.R., Tartof S.Y., Sallis J.F., Sall J., Li Q., Smith G.N., A Cohen D. (2021). Physical inactivity is associated with a higher risk for severe COVID-19 outcomes: A study in 48 440 adult patients. Br. J. Sports Med..

[3] Dixit S. (2020). Can moderate intensity aerobic exercise be an effective and valuable therapy in preventing and controlling the pandemic of COVID-19?. Med. Hypotheses.

[4] World Health Organization (WHO) (2020). Coronavirus Disease 2019 (COVID-19).

[5] Fikenzer S., Uhe T., Lavall D., Rudolph U., Falz R., Busse M., Hepp P., Laufs U. (2020). Effects of surgical and FFP2/N95 face masks on cardiopulmonary exercise capacity. Clin. Res. Cardiol..

[6] Egger F., Blumenauer D., Fischer P., Venhorst A., Kulenthiran S., Bewarder Y., Zimmer A., Böhm M., Meyer T., Mahfoud F. (2021). Effects of face masks on performance and cardiorespiratory response in well-trained athletes. Clin. Res. Cardiol..

[7] Driver S., Reynolds M., Brown K., Vingren J.L., Hill D.W., Bennett M., Gilliland T., McShan E., Callender L., Reynolds E. (2021). Effects of wearing a cloth face mask on performance, physiological and perceptual responses during a graded treadmill running exercise test. Br. J. Sports Med..

[8] Umutlu G., Acar N.E., Sinar D.S., Akarsu G., Güven E., Yildirim I. (2022). COVID-19 and physical activity in sedentary individuals: Differences in metabolic, cardiovascular, and respiratory responses during aerobic exercise performed with and without a surgical face masks. J. Sports Med. Phys. Fit..

[9] Epstein D., Korytny A., Isenberg Y., Marcusohn E., Zukermann R., Bishop B., Minha S., Raz A., Miller A. (2020). Return to training in the COVID-19 era: The physiological effects of face masks during exercise. Scand. J. Med. Sci. Sports.

[10] Shaw K., Butcher S., Ko J., Zello G.A., Chilibeck P.D. (2020). Wearing of Cloth or Disposable Surgical Face Masks has no Effect on Vigorous Exercise Performance in Healthy Individuals. Int. J. Environ. Res. Public Health.

[11] Rosa B.V., Rossi F.E., Moura H., Santos A., Véras-Silva A.S., Ribeiro S.L.G., Nakamura F.Y., dos Santos M.A.P. (2021). Effects of FFP2/N95 face mask on low- and high-load resistance exercise performance in recreational weight lifters. Eur. J. Sport Sci..

[12] Ramos-Campo D., Pérez-Piñero S., Muñoz-Carrillo J., López-Román F., García-Sánchez E., Ávila-Gandía V. (2021). Acute Effects of Surgical and FFP2 Face Masks on Physiological Responses and Strength Performance in Persons with Sarcopenia. Biology.

[13] Smith M.M., Sommer A.J., Starkoff B.E., Devor S.T. (2013). Crossfit-Based High-Intensity Power Training Improves Maximal Aerobic Fitness and Body Composition. J. Strength Cond. Res..

[14] Schlegel P. (2020). CrossFit(R) Training Strategies from the Perspective of Concurrent Training: A Systematic Review. J. Sports Sci. Med..

[15] Heavens K.R., Szivak T.K., Hooper D.R., Dunn-Lewis C., Comstock B.A., Flanagan S.D., Looney D.P., Kupchak B.R., Maresh C.M., Volek J.S. (2014). The Effects of High Intensity Short Rest Resistance Exercise on Muscle Damage Markers in Men and Women. J. Strength Cond. Res..

[16] Domingues W.J.R., Soares A., Cavalcante B.R., Da Silva R.R.M., Nunhes P.M., Da Silva G.M.G., Bezerra E., Oliveira N.L., Oliveira J. (2019). Influence of the order of aerobic and resistance exercise on hemodynamic responses and arterial stiffness in young normotensive individuals. J. Bodyw. Mov. Ther..

[17] Dantas T.S.P., Aidar F.J., de Souza R.F., de Matos Gama D., Ferreira A.R.P., de Almeida Barros N. (2018). Evaluation of a CrossFit[R] Session on Post-Exercise Blood Pressure. J. Exerc. Physiol. Online.

[18] Kingsley J.D., Tai Y.L., Mayo X., Glasgow A., Marshall E. (2017). Free-weight resistance exercise on pulse wave reflection and arterial stiffness between sexes in young, resistance-trained adults. Eur. J. Sport Sci..

[19] Tai Y.L., Gerhart H., Mayo X., Kingsley J.D. (2016). Acute resistance exercise using free weights on aortic wave reflection characteristics. Clin. Physiol. Funct. Imaging.

[20] Shaw S.B., Dullabh M., Forbes G., Brandkamp J.-L., Shaw I. (2015). Analysis of physiological determinants during a single bout of Crossfit. Int. J. Perform. Anal. Sport.

[21] Chao H.-H., Liao Y.-H., Chou C.-C. (2021). Influences of Recreational Tennis-Playing Exercise Time on Cardiometabolic Health Parameters in Healthy Elderly: The ExAMIN AGE Study. Int. J. Environ. Res. Public Health.

[22] Meaney E., Alva F., Moguel R., Meaney A., Alva J., Webel R. (2000). Formula and nomogram for the sphygmomanometric calculation of the mean arterial pressure. Heart.

[23] Ansari M., Javadi H., Pourbehi M.R., Mogharrabi M., Rayzan M., Semnani S., Jallalat S., Amini A., Abbaszadeh M., Barekat M. (2012). The association of rate pressure product (RPP) and myocardial perfusion imaging (MPI) findings: A preliminary study. Perfusion.

[24] Jouven X., Empana J.-P., Schwartz P.J., Desnos M., Courbon D., Ducimetière P. (2005). Heart-Rate Profile during Exercise as a Predictor of Sudden Death. N. Engl. J. Med..

[25] Miller M.R., Hankinson J., Brusasco V., Burgos F., Casaburi R., Coates A., Crapo R., Enright P., Van Der Grinten C.P.M., Gustafsson P. (2005). Standardisation of spirometry. Eur. Respir. J..

[26] Li Y., Tokura H., Guo Y., Wong A., Wong T., Chung J., Newton E. (2005). Effects of wearing N95 and surgical facemasks on heart rate, thermal stress and subjective sensations. Int. Arch. Occup. Environ. Health.

[27] Lässing J., Falz R., Pökel C., Fikenzer S., Laufs U., Schulze A., Hölldobler N., Rüdrich P., Busse M. (2020). Effects of surgical face masks on cardiopulmonary parameters during steady state exercise. Sci. Rep..

[28] Polito M.D., Farinatti P. (2009). The Effects of Muscle Mass and Number of Sets During Resistance Exercise on Postexercise Hypotension. J. Strength Cond. Res..

[29] Simão R., Fleck S.J., Polito M., Monteiro W., Farinatti P. (2005). Effects of Resistance Training Intensity, Volume, and Session Format on the Postexercise Hypotensive Response. J. Strength Cond. Res..

[30] Brito A.D.F., De Oliveira C.V.C., Santos M.D.S.B., Santos A.D.C. (2013). High-intensity exercise promotes postexercise hypotension greater than moderate intensity in elderly hypertensive individuals. Clin. Physiol. Funct. Imaging.

[31] Brito A.D.F., Brasileiro-Santos M.D.S., de Oliveira C.V.C., da Nóbrega T.K.S., Forjaz C.L.D.M., Santos A.D.C. (2015). High-Intensity Resistance Exercise Promotes Postexercise Hypotension Greater than Moderate Intensity and Affects Cardiac Autonomic Responses in Women Who Are Hypertensive. J. Strength Cond. Res..

[32] Cavalcante P.A., Rica R., Evangelista A., Serra A., Junior F.P., Kilgore L., Baker J., Bocalini D., Junior A.F. (2015). Effects of exercise intensity on postexercise hypotension after resistance training session in overweight hypertensive patients. Clin. Interv. Aging.

[33] Coelho-Júnior H.J., Irigoyen M.-C., Aguiar S.D.S., Gonçalves I.D.O., Câmara N.O.S., Cenedeze M.A., Asano R.Y., Rodrigues B., Uchida M.C. (2017). Acute effects of power and resistance exercises on hemodynamic measurements of older women. Clin. Interv. Aging.

[34] Li Y., Bopp M., Botta F., Nussbaumer M., Schäfer J., Roth R., Schmidt-Trucksäss A., Hanssen H. (2015). Lower Body vs. Upper Body Resistance Training and Arterial Stiffness in Young Men. Endoscopy.

[35] Shein S.L., Whitticar S., Mascho K.K., Pace E., Speicher R., Deakins K. (2021). The effects of wearing facemasks on oxygenation and ventilation at rest and during physical activity. PLoS ONE.

[36] Albiol L.A., Paya N.S., Garnacho-Castaño M.A., Cano L.G., Cobo E.P., Maté-Muñoz J.L., Garnacho-Castaño M.V. (2019). Ventilatory efficiency during constant-load test at lactate threshold intensity: Endurance versus resistance exercises. PLoS ONE.

[37] Ohya T., Yamanaka R., Hagiwara M., Oriishi M., Suzuki Y. (2016). The 400- and 800-m Track Running Induces Inspiratory Muscle Fatigue in Trained Female Middle-Distance Runners. J. Strength Cond. Res..

[38] Chevrolet J.C., Tschopp J.M., Blanc Y., Rochat T., Junod A.F. (1993). Alterations in inspiratory and leg muscle force and recovery pattern after a marathon. Med. Sci. Sports Exerc..

[39] Ozkaplan A., Rhodes E.C., Sheel A.W., Taunton J.E. (2005). A comparison of inspiratory muscle fatigue following maximal exercise in moderately trained males and females. Eur. J. Appl. Physiol..

[40] Gonzales J.U., Williams J.S. (2010). Effects of acute exercise on inspiratory muscle strength and endurance in untrained women and men. J. Sports Med. Phys. Fit..

[41] Poon E.T.-C., Zheng C., Wong S.H.-S. (2021). Effect of Wearing Surgical Face Masks During Exercise: Does Intensity Matter?. Front. Physiol..

